# SOST and DKK: Antagonists of LRP Family Signaling as Targets for Treating Bone Disease

**DOI:** 10.4061/2010/460120

**Published:** 2010-07-01

**Authors:** James J. Mason, Bart O. Williams

**Affiliations:** ^1^Center for Skeletal Disease Research, Van Andel Research Institute, 333 Bostwick NE, Grand Rapids, MI 49503, USA; ^2^Laboratory of Orthopaedic Cell and Tissue Mechanics, Van Andel Research Institute, 333 Bostwick NE, Grand Rapids, MI 49503, USA; ^3^Laboratory of Cell Signaling and Carcinogenesis, Van Andel Research Institute, 333 Bostwick NE, Grand Rapids, MI 49503, USA

## Abstract

The study of rare human genetic disorders has often led to some of the most significant advances in biomedical research. One such example was the body of work that resulted in the identification of the Low Density Lipoprotein-Related Protein (LRP5) as a key regulator of bone mass. Point mutations were identified that encoded forms of LRP5 associated with very high bone mass (HBM). HBM patients live to a normal age and do not appear to have increased susceptibility to carcinogenesis or other disease. Thus, devising methods to mimic the molecular consequences of this mutation to treat bone diseases associated with low bone mass is a promising avenue to pursue. Two groups of agents related to putative LRP5/6 functions are under development. One group, the focus of this paper, is based on antagonizing the functions of putative inhibitors of Wnt signaling, Dickkopf-1 (DKK1), and Sclerostin (SOST). Another group of reagents under development is based on the observation that LRP5 may function to control bone mass by regulating the secretion of serotonin from the enterrochromaffin cells of the duodenum.

## 1. Introduction

During the last decade, several groups working on the genetics of rare human skeletal disorders observed that mutations in what were thought to be core or regulatory components of the Wnt/*β*-catenin pathway lead to dramatic phenotypic effects. These mutations were either in the gene encoding the low density lipoprotein receptor-related protein-5 (*LRP5*) or in a gene (*SOST*) encoding a protein (Sclerostin) that potentially binds and regulates the function of LRP5 and its family members LRP4 and LRP6. This work has established LRP5 as a major target for drug development to treat osteoporosis and other bone diseases. This paper will discuss the development of two groups of agents designed to activate LRP5 (and the related LRP6) signaling pathway to increase bone mass. We will first review the core components of the Wnt signaling pathway to put the development of these agents into a cellular context.

These are by no means the only agents related to the identification of LRP5 that are in clinical development. One of the more interesting areas of research has centered around the observation by Yadav and colleagues that loss of LRP5 leads to low bone mass due to dysregulation of serotonin synthesis from the enterochromaffin cells of the duodenum [1]. Normally, LRP5 inhibits the expression of TPH1, the rate-limiting enzyme for serotonin production in enterochromaffin cells. In the absence of LRP5 in both humans and mice, serum serotonin levels were reported to rise and act on the HTR1B receptor in osteoblasts to inhibit their proliferation [1, 2]. Patients and mice carrying alleles of LRP5 associated with high bone mass are reported to have decreased levels of serum serotonin [1, 3]. This has led to the exciting possibility that pharmacological modulation of serum serotonin levels could be an effective treatment for low bone mass, a possibility supported by a recent report. More detailed discussions of this potential treatment can be found in several recent reviews [4, 5].

While not discounting the potential importance of serotonin-based therapies, this paper primarily focuses on the development of agents that potentially target LRP5, and the related proteins LRP6 and LRP4, in the bone itself.

## 2. Overview of Wnt Signaling

**Figure 1:**
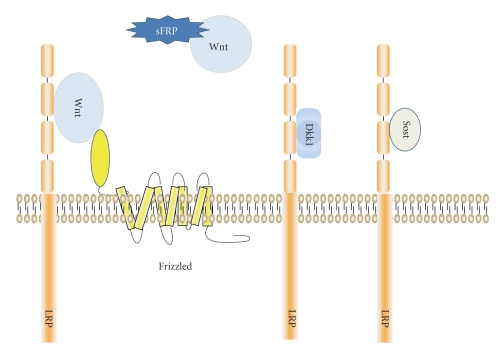
The current model for the induction of stabilization of *β*-catenin by Wnt ligands holds that Wnt proteins bind a complex that includes a member of the frizzled family of seven-transmembrane-spanning receptors and either LRP6 or LRP5. Several proteins have been identified that can block this process and are associated with downregulation of Wnt signaling. These include proteins which bind to the LRP component to prevent association of Wnt ligands with the LRPs (Dkk1 and Sost). In addition, secreted frizzled related proteins (sFRPs) also can block signaling by binding directly to Wnt ligands and potentially interfering with their ability to engage the receptor complex.

Mammals contain 19 genes encoding Wnt ligands. Wnts are cysteine-rich, glycosylated, and lipid-modified proteins that are highly associated with the extracellular matrix, particularly heparin sulfate glycoproteins [6]. Wnts can activate several signaling cascades, including one that results in the stabilization of *β*-catenin in the cytoplasm followed by its nuclear localization [7]. Wnts initiate signaling by binding to a member of the Frizzled family of seven transmembrane receptors and either LRP5 or LRP6 [8–10] (Figure 1), leading to downregulation of glycogen synthase kinase-3 (GSK-3) activity. In the absence of Wnt ligands, GSK-3 phosphorylates *β*-catenin on residues near it amino terminal end, marking it for ubiquitin-dependent proteolysis [11]. Inactivation of GSK-3 increases *β*-catenin levels in the cytosol. Recent work has also uncovered a parallel set of signals that are initiated by activation of the Wnt receptor complex that lead to the phosphorylation of *β*-catenin on serine residues (in regions C terminal to and independent from the GSK3 sites). Phosphorylation of these residues is required for efficient nuclear translocation [12, 13]. The combined effect of increasing levels of cytosolic *β*-catenin and facilitating its translocation to the nucleus allows *β*-catenin to form complexes with members of the Tcf/Lef class of DNA binding proteins [14]. These complexes modulate transcriptional activity of target promoters [14].

This core pathway is regulated by a large number of extracellular and intracellular proteins. Extracellular proteins that interact with this pathway include members of the Dickkopf family and Sclerostin and secreted frizzled related proteins (sFRPs) which regulate signaling at the level of the Wnt/Frizzled/Lrp interaction [15–21] (Figure 1). In addition, many proteins (including GBP/FRAT, axin, *β*TrCP, and APC, the product of the *adenomatous polyposis coli* tumor suppressor gene) control the pathway by regulating components of the intracellular signaling pathway [9, 14].

The effects of regulating GSK3 activity by Wnt signaling can also directly activate the mammalian target of rapamycin (mTOR) pathway by decreasing GSK-3-mediated activation of the TSC2/TSC1 complex [22]. This observation extends our understanding of the role of Wnt signaling in cellular regulation and identifies mTOR as an important downstream affector of Wnt signaling and, by extension, a potential downstream target of Lrp5 and/or Lrp6 during osteoblast differentiation. Activation of the mTOR pathway by Wnt ligands is independent of *β*-catenin, highlighting a signaling cascade that could explain different phenotypes seen when the pathway is inactivated at the level of Lrp5 and/or Lrp6 compared to inactivation of *β*-catenin. In addition to the canonical pathway [14], other signaling cascades initiated by Wnts include pathways that signal through the Rho GTPases and calcium-dependent pathways [23]. For more detailed descriptions of the Wnt signaling pathway, several excellent recent reviews on the subject are available [6–8, 24–27].

## 3. Overview of LRP Family Members

Although partially redundant, Lrp4, Lrp5, and Lrp6 display clearly distinct functions. For example, Lrp6-deficient mice die at birth [28], whereas Lrp5- and Lrp4-deficient mice are viable [29], suggesting unique functions of these receptors that cannot be compensated for by the others. While Lrp5-deficient mice develop a normal skeletal structure [30], Lrp6-deficient mice exhibit long bone formation defects. These defects are reminiscent of those observed in Wnt-7a and Wnt-1 mutant mice, indicating a possible link between Wnt1 or Wnt7a and Lrp6 that may not exist between these Wnts and Lrp5 [28]. Another possibility is that the role of Lrp6 may also involve down regulation of the Wnt5a noncanonical signaling pathways.

It was recently shown that Lrp6 physically interacts with Wnt5a, but that this does not lead to phosphorylation of Lrp6 or activation of the Wnt/*β*-catenin pathway. Overexpression of Lrp6 blocks activation of the Wnt5a-target, Rac, and this effect is dependent on intact Lrp6 extracellular domains. Surprisingly, some Lrp6−/− birth defects were rescued by deletion of Wnt5a, indicating that the phenotypes resulted from noncanonical Wnt gain-of-function [31]. Finally, the Wnt5a loss-of-function birth defect is consistent with Ca2^+^ modulation having an antagonistic interaction with Wnt/*β*-catenin signaling [32]. 

Similar to Lrp6, Lrp4 (a.k.a. Megf7) plays a role in limb development [33]. Lrp4-deficient mice have less severe phenotypes than those lacking Lrp6, but more severe than Lrp5-deficient mice. Phenotypes in Lrp4-deficient mice include a fully penetrant form of polysyndactyly and a mild and incompletely penetrant form of craniofacial abnormalities [34]. 

Detailed analysis of the functions of these receptors in several additional tissues using several mouse models are in progress. These include the mammary gland, where the functions of both Lrp5 and Lrp6 are linked to mammary progenitor cell regulation and where the proteins appear to function in at least a partially redundant fashion and affect the levels of Wnt/*β*-catenin signaling within the mammary gland [35–37]. In addition, changes in both LRP5 and LRP6 have been linked to alterations in glucose homeostasis and lipid metabolism [38–43], although it is not certain whether these latter functions are dependent on the Wnt/*β*-catenin signaling pathway.

Recent studies have also highlighted an important unanswered question related to the function of LRP family receptors in osteoblasts. Conditional deletion of *β*-catenin, or activation of the pathway by either inducible expression of a oncogenic version of *β*-catenin or via deletion of the Apc gene, leads to dramatic effects on osteoclastogenesis due to altered regulation of osteoprotegerin expression [44, 45]. In contrast, neither humans nor mice lacking LRP5 display any apparent alteration in osteoclast differentiation or function. There are several potential explanations for this. One is that the functions of LRP5 within the duodenum may play a predominant role [1]. It is also possible that LRP6 and LRP5 play redundant roles in regulating this process [46]. These possibilities are being actively examined by several laboratories.

## 4. Linkage of LRP5 Mutations to Conditions with Altered Bone Mass

During the early part of this past decade, several reports linked changes in bone mass to alterations in *LRP5*. The first report found that patients with osteoporosis pseudoglioma syndrome (OPPG), an autosomal recessive disorder in which afflicted individuals develop severe, early-onset osteoporosis [47], are homozygous for inactivating mutations in *LRP5 * [48]. These individuals have a very high susceptibility to multiple fractures and have severe deficits in vision due to persistence of the hyaloid vasculature often associated with retinal detachment [47]. Shortly after loss of LRP5 function was linked to OPPG, two groups independently reported that families with extremely high bone mass (HBM) carried a specific point mutation (G171V) in *LRP5 * [49, 50]. The LRP5-G171V protein can no longer be bound by several proteins (such as Dkk1, Sost, and MESD) that may normally regulate its activity. Subsequently, work in mouse models by several laboratories provided further confirmation for a role of *Lrp5* in regulating bone mass [30, 46, 51–56].

LRP5 is a member of a multigene family and several other members of this family have shown to be involved in bone development and disease. For example, mutations in *LRP6, *which shares greater than 70% identity with LRP5, have been linked to changes in bone mass in both humans and mice [28, 30, 53, 56, 57]. In addition, it has recently been shown that *LRP4*, which is expressed in bone and cultured osteoblasts, binds Dkk1 and sclerostin in vitro and that *Lrp4*-deficient mice revealed shortened total femur length, reduced cortical femoral perimeter, reduced total femur bone mineral content (BMC), and bone mineral density (BMD) [58]. Thus, Lrp4 is also an osteoblast-expressed Dkk1- and sclerostin-receptor with a physiological role in the regulation of bone growth and turnover.

While it is important to note that some HBM patients develop pain neurologic sequelae [59], the fact that these patients do not appear to have an obvious predisposition to cancer or other disease has led to several biotechnology and pharmaceutical companies investing large amounts of resources in developing agents in an attempt to mimic the effects of the LRP5 mutations associated with HBM [60]. Since the canonical Wnt pathway is ubiquitous in embryonic development and oncogenesis [7], targeting LRP4-6 directly may have unintended effects. For example, both *Lrp4* and *Lrp6*-deficient mice exhibit significant developmental deformities [28, 33]. However, by concentrating on agonists or antagonists specific to bone, we may significantly reduce those risks. Here, we focus on two groups of agents; those designed to inhibit the function of Sclerostin and those which block the activity of the DKK1 protein. Further, we briefly discuss sFRPs, whose affects on bone are only recently being reported.

## 5. Sclerosteosis and Van Buchem's Disease

Sclerosteosis is autosomal recessive disorder characterized by progressive skeletal overgrowth [61, 62]. Patients appear normal at birth, with the exception of some instances of syndactyly. Skeletal overgrowth, especially in the mandible and skull, commences early in life. This can cause compression of the 7th and 8th cranial nerves often resulting in facial palsy and conductive hearing loss.

In 2001, it was reported that a gene located on Chromosome 17q11.2 was mutated in sclerosteosis [63, 64]. This gene which encodes a secreted glycoprotein (Sclerostin or Sost) containing a cysteine knot-like domain with homology to the Cerebrus/DAN family of BMP antagonists [63, 64]. Subsequent work on a related disorder, Van Buchem's Disease, revealed that while there was no mutation in the coding region of the *SOST* gene, a homozygous 52 kB deletion in a region closely linked to the *SOST* gene was identified in these patients [65–67]. Patients with Van Buchem's display what is essentially a milder version of the symptoms observed in Sclerosteosis. Additional work suggests that this deletion results in downregulation of Sost expression [65–67].

Partly due to its homology to Cerberus and DAN family members, it was originally thought that loss of Sost lead to bone abnormalities primarily due to ectopic activation of BMP pathways [68, 69]. However, subsequent work demonstrated that it also bound Lrp5 and Lrp6 and could prevent their interaction with Wnts [17–19]. Thus, loss of Sost may lead to an inability to inactivate the Wnt signaling pathway. Consistent with the skeletal overgrowth seen in patients carrying the G171V mutation in LRP5, Sost is unable to interact with the mutated version of LRP5 [70, 71].

A key characteristic that makes Sost a particularly attractive target for the treatment of osteoporosis is that its expression is restricted to osteocytes [72]. Thus, unintended side effects caused by blocking activity of this protein in other tissues are less likely. Furthermore, genetically engineered mouse models designed to mimic the mutations seen in Sclerosteosis and van Buchem's patients accurately model the high bone mass changes seen in humans [73].

Based on these characteristics, several pharmaceutical companies have initiated programs to create biological agents that inhibit Sost activity. Amgen, Novartis, and Eli Lilly have all been reported to have developed monoclonal antibodies designed to inhibit SOST [74]. In addition, OsteoGeneX has reportedly developed a small molecule inhibitor of SOST that is in the preclinical development stage [74].

Evidence for the potential efficacy of such approaches has been found in at least two preclinical models. Amgen reported that an antibody that blocked SOST function increased bone formation, bone strength, and bone mass in a rat model of postmenopausal osteoporosis [75]. Furthermore, a similar antibody was reported to inhibit bone loss in a mouse model of chronic colitis [76].

## 6. Dickkopf 1

Dickkopf1 (DKK1) is the prototype of a 4 member gene family and was first identified in 1998 [77]. Dkk proteins contain two cysteine-rich domains. The more N-terminal domain is Dkk-family specific, while the second domain contains structural homology to the colipase fold [77]. At that time, it was reported to be a secreted protein that inhibited Wnt signal transduction, but did not bind directly to Wnt proteins. 

After LRP5 and LRP6 were identified as putative coreceptors for Wnt ligands [28, 78, 79], several groups reported that Dkk1 inhibited Wnt/*β*-catenin signaling via binding directly to LRP5 and LRP6 and blocked the ability of Wnt ligands to interact with LRP5 and LRP6 [80–82]. In addition, some reports (but not all [71]) found that the version of the LRP5 protein found in HBM families (G171V) could not bind to or be inhibited by DKK1 [83, 84]. Subsequent work showed that three members of this family (DKK1, DKK2, and DKK4) were inhibitors of the Wnt/*β*-catenin pathway, while DKK3 was divergent in both function and structure [77].

Mouse models have provided further support for a key role for *Dkk1* in bone development. Germline deficiency for *Dkk1* results in embryonic lethality associated with absence of head structures anterior to the midbrain and abnormalities of digits in the limbs [85]. Studies of mice heterozygous for an inactivating mutation in *Dkk1* show high bone mass associated with a significant increase in the bone formation rate [86]. In addition, heterozygosity for a hypomorphic allele of *Dkk1* (doubleridge) also results in increased bone mass [87].

Based on these observations, several companies are pursuing therapeutic approaches for bone disease based on inactivating Dkk1 function. These include Nuvelo's development of a monoclonal antibody against Dkk1 and a small molecule inhibitor approach being pursued by Enzo Biochem [74]. To our knowledge, there has not been published evidence for efficacy studies in the area of osteoporosis. However, several studies have found anti-Dkk1 antibodies were effective in treating disease on preclinical modeling systems. For example, administration of such an antibody immediately following a fracture significantly enhanced bone repair [88]. There are also several examples of anti-Dkk1 antibodies modulating the severity of diseases such as multiple myeloma and osteoarthritis [89–91].

## 7. Secreted Frizzled Related Proteins

Secreted frizzled related proteins are similar to DKK1 and Sclerostin in that they also inhibit Wnt/*β*-catenin signaling. However, they do so through a different molecular mechanism. sFRPs inhibit canonical Wnt signaling by binding directly to the Wnt molecule itself [92, 93]. sFRPs share sequence similarity with the cysteine-rich domain (CRD) found in the extracellular region of frizzled. sFRPs bind the Wnt ligands through their CRD, thereby preventing their binding to Frizzled receptors [94]. Results related to the effects of sFRPs are fairly recent. To date, sFRP-1 has been shown to be involved in the anabolic affects of PTH; deletion of sFRP-1 resulted in increased trabecular bone mineral density in a mouse model [95, 96]. Furthermore, sFRP-2 has been shown to inhibit bone formation [97]. It should be noted, though, that down regulation of sFRP-1 predisposes the mammary gland to tumorigenesis [98] while sFRP-2 is significantly downregulated in gastric cancer [99], and downregulation of both sFRP-1 and sFRP-2 contributes to cervical cancer progression [100]. Thus, approaches aimed at inactivating sFRP function [95, 101, 102] should be pursued with appropriate caution. Similar risks exist with DKK1 [103] and Sclerostin [104]. However, research to date has not shown any predisposition of treatments utilizing these molecules toward oncogenesis (see below).

## 8. Future Directions

Preclinical studies with agents designed to block the functions of Sost and DKK1 have shown promise in treating bone disease and will likely be soon entering human clinical trials for the treatment of osteoporosis. Ongoing work will undoubtedly identify other potential druggable targets within this pathway. For example, the finding that the Prorenin receptor acts as an rennin-independent adaptor between LRP6 (and potentially LRP5) and the vacuolar H^+^–adenosine triphosphatase (V-ATPase) complex may provide new drug targets [105]. The subsequent acidification of this compartment is required for phosphorylation of the cytoplasmic tail of LRP6, which is necessary for activating the downstream signaling cascade [106–108]. One could envision approaches designed to enhance this event to increase Wnt signaling and bone mass.

In addition to the development of agents directly targeting components of this pathway, several current and potential treatments for low bone mass may interact with the Wnt signaling pathway. For example, the anabolic actions of Parathyroid hormone (the basis for Forteo/teriparatide [109]) have been proposed to directly and/or indirectly work through regulation of LRP6 and/or LRP5 signaling [110–112]. In addition, osteoprotegerin (OPG), a molecule produced by cells of the osteoblast lineage that inhibits activation of osteoclasts [113], is a direct transcriptional target for *β*-catenin [44, 45]. Denosumab, a monoclonal antibody in clinical trials developed by Amgen [114], is based on the function of OPG. However, while the regulation is clearly altered in mice carrying mutations which directly activate or inactivate *β*-catenin, it does not appear to be altered in mice or humans carrying inactivating mutations in* LRP5 * [48, 51]. This demonstrates the potential complexity of regulation within these pathways, and emphasizes the critical need to increase our knowledge about the detailed regulation of Wnt signaling pathways within osteoblasts.

Activation of the Wnt signaling pathway is one of the most common events associated with human cancer [7, 115]. As previously noted, the potential for treatments aimed at activating the Wnt signaling pathway to increase bone mass must always been tempered by consideration of a potentially increased susceptibility to carcinogenesis or other deleterious consequences. However, several observations suggest that this may not be as large a concern in the context of treating bone disease as some originally feared. First, neither the LRP5 HBM patients nor those with Sclerosteosis are reported to have an increased rate of carcinogenesis. In addition, there have been no reports in the preclinical modeling studies of increased carcinogenesis in mice treated with agents that block Dkk1 or Sost. Finally, lithium chloride has been used for decades to treat psychiatric illnesses in humans without being associated with any apparent increase in cancer risk. Given that the main mechanism of action for lithium treatment is inhibition of GSK3 activity (associated with upregulation of *β*-catenin signaling) [116], this provides further confidence in the approaches discussed in this paper.

Some concerns should also exist regarding the effects of systemic upregulation of Wnt/*β*-catenin signaling on fracture healing. While it is presumable that efficacy of these drugs would reduce fragility fractures in osteoporotic patients, the likelihood of fracture due to moving vehicle accidents or other misfortune is not necessarily reduced. Therefore, the effects of potential drugs on healing cannot be ignored. Unfortunately, the role of Wnt/*β*-catenin signaling in fracture healing is only beginning to be understood and therefore could lead to difficulties. For example, nonsteroidal antiinflammatory drugs (NSAIDs) that inhibit inflammatory response through down regulation of Cox2 were expected to have little effect on bone healing since mice lacking Cox2 form normal skeletons. However, it was later shown that fracture healing failed in rats treated with COX-2–selective NSAIDs and consequently, it was concluded that COX-2 function is specifically essential for fracture healing but not embryonic skeletal development [117]. Early indications are that Wnt signaling is both upregulated and downregulated temporally throughout the healing process [118]. As such, regulation of canonical Wnt signaling during this process is presently unpredictable. Further investigation into the role of canonical Wnt signaling in fracture healing is required.

In summary, the discovery almost a decade ago that mutations in LRP5 were causally associated with alterations in bone mass has stimulated numerous lines of research that have identified a number of promising targets to treat osteoporosis. The next decade will undoubtedly see the further translation of these findings into clinical use.
